# Targeting gut-brain-immune axis in amyotrophic lateral sclerosis

**DOI:** 10.3389/fimmu.2025.1637976

**Published:** 2026-01-29

**Authors:** Naga Sriharsha Mudda, Lucas Zhang, Pooja Sampelli

**Affiliations:** Student Inquiry and Research, Illinois Mathematics and Science Academy, Aurora, IL, United States

**Keywords:** amyotrophic lateral sclerosis, microbiome, gut-brain axis, neurodegeneration, immune system, inflammation, nicotinamide, SCFAs

## Abstract

Amyotrophic lateral sclerosis (ALS) is a fatal motor neuron neurodegenerative disorder with a median survival of only 3–5 years. The heterogeneity of the disease and lack of effective therapies highlight the importance of identifying novel pathogenic mechanisms. We hypothesize that dysbiosis of gut microbiota enhances ALS by disrupting intestinal barrier function and altering metabolite profiles to drive systemic inflammation and neuronal stress. Precisely, the decrease in health-promoting bacteria (e.g., *Akkermansia muciniphila, Bifidobacterium* and *Lactobacillus spp*.) in ALS can reduce neuroprotective metabolite production (short-chain fatty acids, nicotinamide, GABA, precursors of serotonin) and increase gut permeability, enabling lipopolysaccharide (LPS) and pro-inflammatory cytokines into the circulation. Such changes would activate microglia and impair motor neuron homeostasis by glutamate excitotoxicity and mitochondrial dysfunction. The gut-brain axis operates through immune-mediated mechanisms, where ALS-associated microbiota changes compromise mucosal immunity and trigger peripheral Th1/Th17-biased responses with impaired Treg regulation. Elevated endotoxin levels correlate with TLR4-driven inflammation, promoting pro-inflammatory cytokines (IL-1β, IL-6, TNF-α) that cross into the CNS and prime microglia toward a neurotoxic M1 phenotype, creating a milieu where IL-17A and other mediators directly injure motor neurons. Our hypothesis relies on establishing human and animal evidence of microbiome derangements, barrier dysfunction, and immune deregulation with ALS. We hypothesize that restoration of an “ALS-protective” microbiota consortium or its metabolic by-products can potentially slow disease progression. Testable hypotheses include improvement of ALS model motor deficits by probiotic or fecal-microbiota therapies, and normalization of inflammatory biomarkers. This paradigm recontextualizes ALS as a gut-brain disease and suggests new directions for translational research into this unmet medical indication.

## 1 Introduction

**Figure 1:**
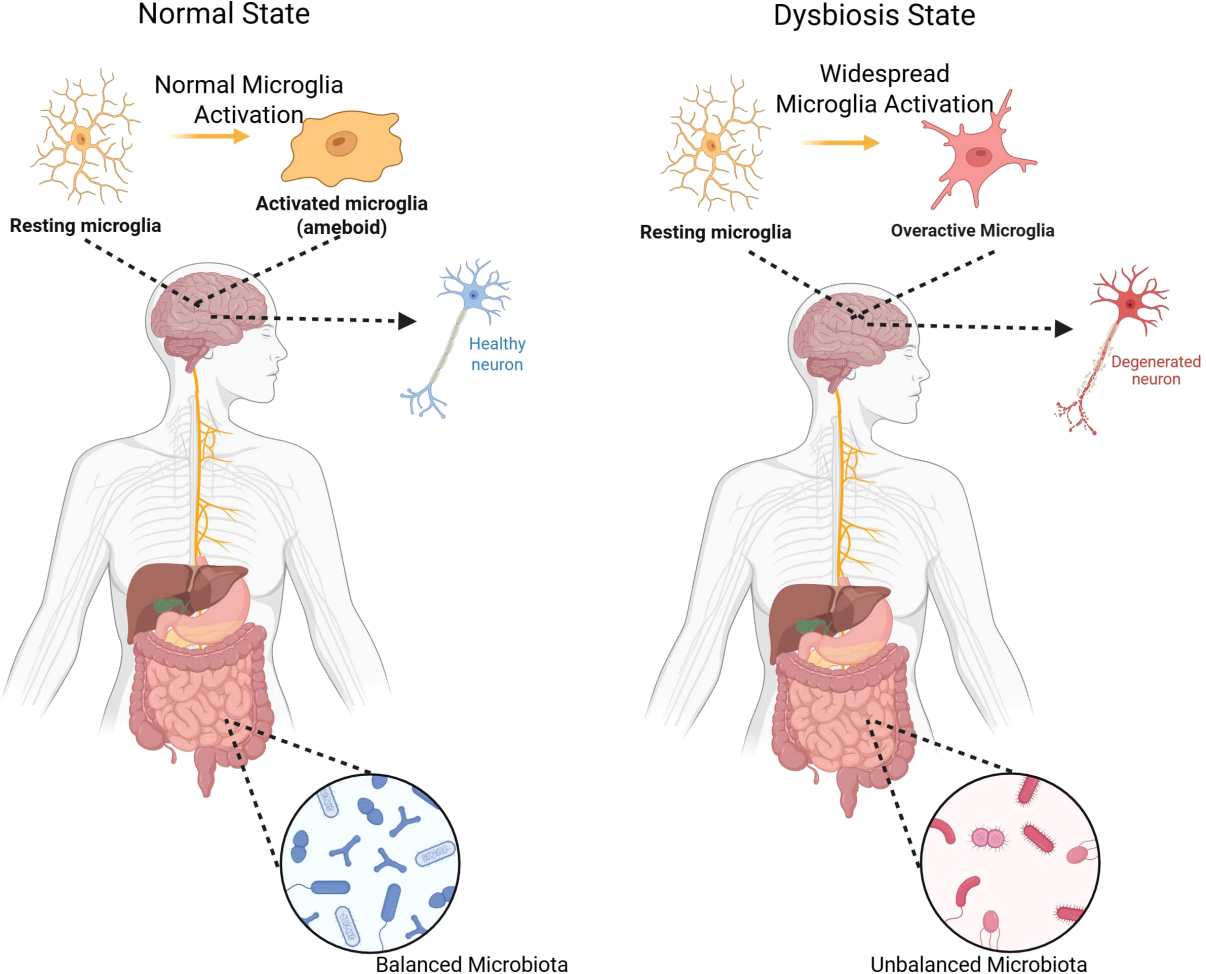
Normal vs. dysbiosis states of the gut microbiome and their effects on microglial activation and neuroinflammation. Healthy microbiota maintain epithelial integrity and anti-inflammatory tone, whereas dysbiosis promotes barrier disruption, systemic inflammation, and neurodegeneration.

Amyotrophic lateral sclerosis (ALS) is a progressive and often fatal neurodegenerative disease of upper and lower motor neurons (1, 2). The vast majority of patients die within 3–5 years from the onset of symptoms, generally to respiratory failure, and no treatment to date has meaningfully changed its inexorable course (1, 2). This sudden unmet need provokes research into unusual pathogenic pathways (3). Emerging evidence implicates the gut-brain axis—the two-way communication between the gut and central nervous system—as a new culprit in ALS (4). Mechanically, the gut micriobiota can influence the pathogenesis of ALS through interconnected pathways, despite the gut and brain being anatomically different structures, specifically through the modulation of systemic and CNS-specific immune responses, communication through the vagus nerve and enteric nervous system (ENS), and neuroendocrine signaling like hormones (5). The microbiota in the gut is also capable of producing or stimulating host production of neurotransmitters such as serotonin, dopamine, and gamma-aminobutyric acid (GABA) (5). In other neurodegenerative disorders (Parkinson’s and Alzheimer’s disease), dysbiosis of the gut has been shown to exacerbate disease by induction of inflammation (6, 7), leaky gut, and altered microbial metabolites (8, 9). Likewise, ALS patients and animal models exhibit atypical microbiota changes (e.g., reduced *Akkermansia* and other commensals) (10–13) and signs of impaired gut integrity (12) Compared to healthy controls, stool samples of ALS patients show less microbial diversity in the gut and signs of intestinal inflammation, creating disbyosis (12). For example, SOD1-G93A ALS mice show early loss of butyrate-producing microbes and intestinal tight-junction proteins, and sodium butyrate supplementation restores their barrier function (10, 13). Broad-spectrum antibiotics or germ-free status, on the other hand, worsens neurodegeneration in these models (14). These findings suggest that a healthy microbiome generally inhibits neurotoxic inflammation and supports motor neuron survival (4, 15), whereas dysbiosis can trigger pathological gut-brain signaling, by affecting pro-inflammatory mediators in the CNS (12, 16) (**Figure 1**).

## 2 Hypothesis

**Figure 2:**
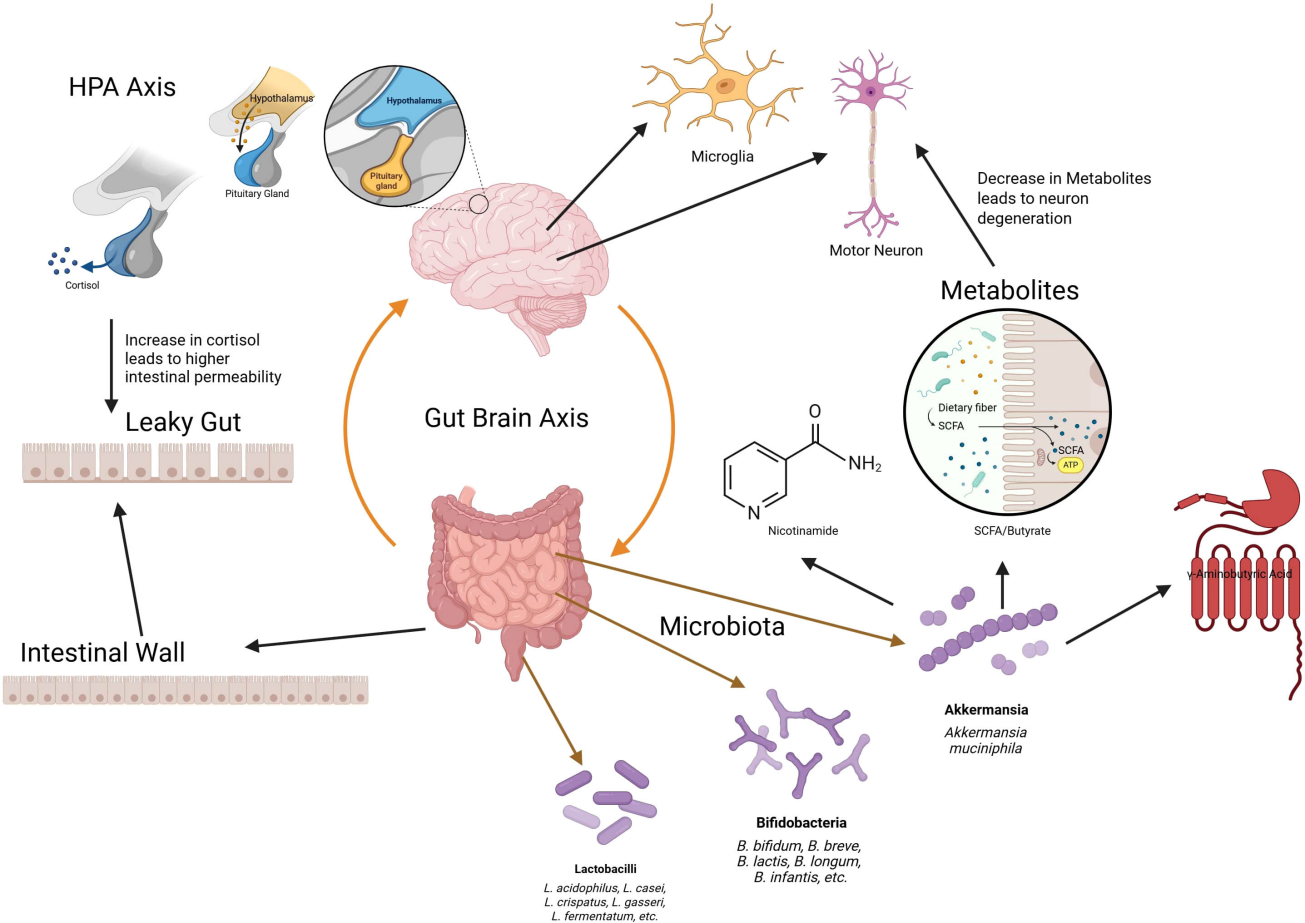
Schematic of the gut–brain axis in ALS. Loss of beneficial microbes reduces neuroprotective metabolites (short-chain fatty acids, tryptophan derivatives), impairs barrier integrity, and increases systemic inflammation. These signals propagate via immune, vagal, and endocrine pathways to exacerbate motor neuron degeneration.

We predict that a dysbiotic gut-microbiome signature triggers a pro-inflammatory, excitotoxic cascade in ALS (10, 11, 13, 17) (**Figure 2**). Specifically, the loss of beneficial microbes (e.g. *Akkermansia muciniphila*, *Bifidobacterium* spp., *Lactobacillus* spp.) leads to the following: impaired mucus and epithelial barriers (12, 18), reduced production of neuroprotective metabolites (e.g., SCFAs, nicotinamide, *γ*-aminobutyric acid (GABA), serotonin precursors) (9, 10, 19), and augmented translocation of pro-inflammatory factors (LPS, microbial amyloids) into the bloodstream (18, 20). Such changes evoke innate immunity, activate peripheral T cells, and microglia (11, 20) and disrupt glutamate-GABA equilibrium in the CNS (10, 13, 21), causing damage to motor neurons.

This points to three ALS mechanisms: systemic inflammation via cytokines/LPS (3, 18, 22), glutamate excitotoxicity via deprivation of GABA/serotonin modulation (10, 13), and mitochondrial failure via NAD deficit (10). Perturbations in the gut microbiota have been shown to alter the composition of circulating cytokines and chemokines, including immune mediators capable of crossing the blood-brain barrier and activating microglia (14). Such shifts in peripheral immune tone can reshape microglial function and morphology, promoting neuroinflammatory states that accelerate neuronal damage (14). In contrast, a healthy microbiota delivers butyrate and nicotinamide to maintain blood-brain barrier function and mitochondrial metabolism (10, 11, 13), while also delivering GABA and serotonin to mitigate excitotoxicity (9, 23). We thus hypothesize a causative link in which disruption of the microbiome leads—or contributes—to ALS, offering a tractable target for intervention (4, 14, 15, 24).

## 3 Supporting evidence

### 3.1 Gut microbiota changes in ALS

Several human cohort studies have described dysregulated gut communities in ALS, although the evidence is inconsistent. A 2023 study of ALS patients identified various microbial alterations: elevated Enterobacter, Clostridium, Veillonella and reduced Prevotella, Lactobacillus and other SCFA-producers compared to controls (11, 25). Some research identifies a reduced Firmicutes: Bacteroidetes ratio in ALS patients, while others find no difference (4), again attributing cohort heterogeneity (13). Notably, a large Swedish study observed that high antibiotic exposure (which disrupts gut flora) was associated with higher ALS risk (12, 26). In SOD1-G93A mice, there is dysbiosis before symptom onset, with the disappearance of Butyrivibrio and other butyrate-producers (13). Major human colonic butyrate producers include Faecalibacterium prausnitzii, Eubacterium rectale/Eubacterium hallii (Anaerobutyricum hallii), Roseburia spp., Butyrivibrio spp., and Anaerostipes spp., predominantly within Clostridium clusters IV and XIVa, with cross-feeding from Bifidobacterium-derived acetate and lactate sustaining butyrate output *in vivo* (8, 11, 13). Together, these results establish an ALS-associated gut dysbiosis (12), albeit with small sample sizes and confounders (diet, disability) preventing causality from being proven (11).

### 3.2 Barrier integrity and inflammation

**Figure 3:**
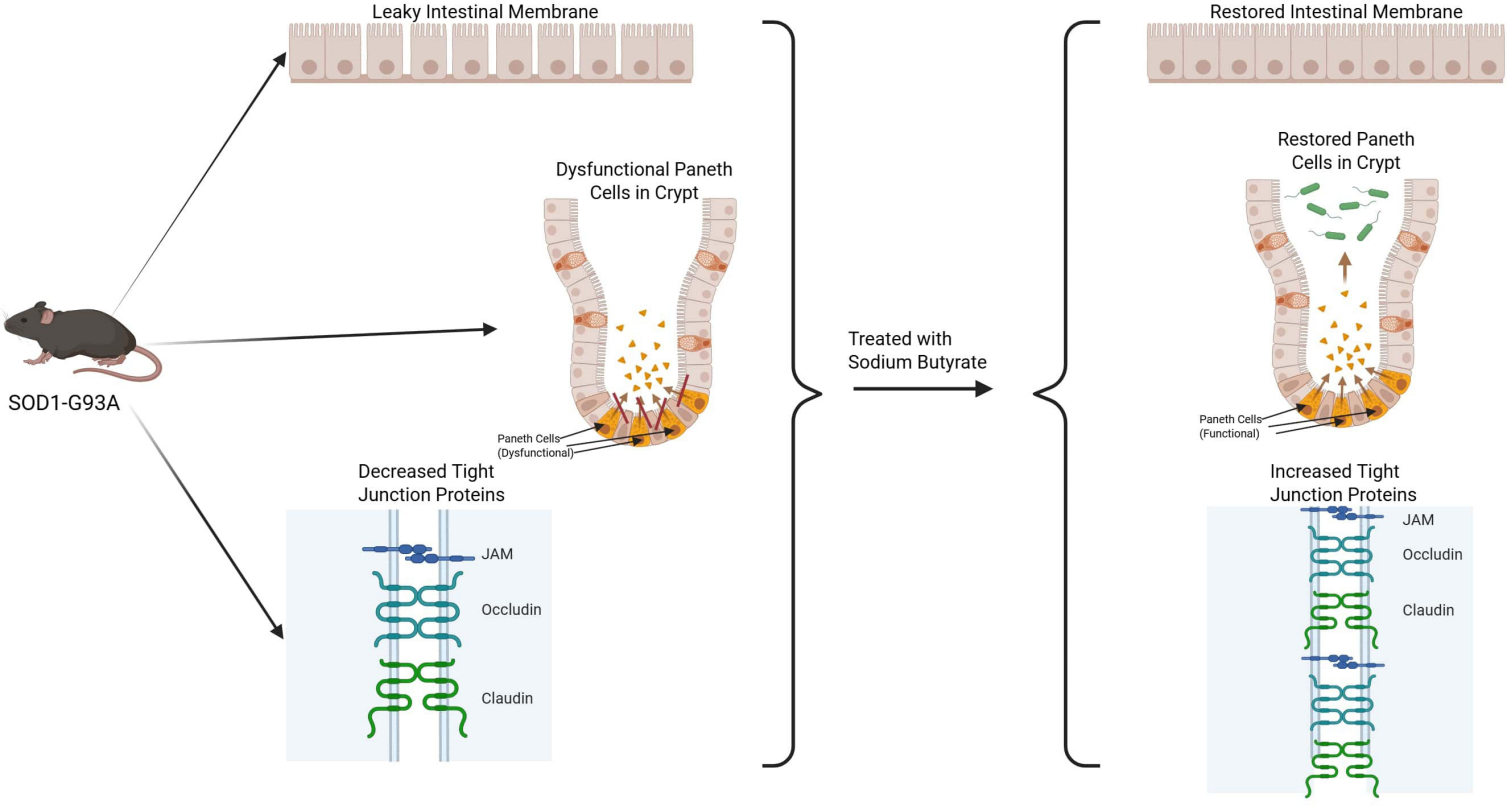
Evidence from ALS mouse models. In SOD1-G93A mice, gut dysbiosis correlates with reduced tight-junction proteins and intestinal barrier breakdown. Sodium butyrate supplementation restores barrier function and slows neurodegeneration.

Abnormal gut barriers have been demonstrated in ALS models: SOD1-G93A mice have leaky intestines, dysfunctional Paneth cells, and reduced tight-junction proteins (13). Treatment with sodium butyrate in these mice restored barrier function and retarded neurodegeneration (13). Elevated intestinal permeability markers (serum zonulin, DAO) and circulating LPS have also been reported in some ALS patients, indicating microbial translocation (3). Translocated LPS could activate toll-like receptors on microglia and induce systemic cytokine release (20), linking gut leakiness to the chronic neuroinflammatory state in ALS (20). Supporting this, antibiotic gut microbiome elimination in SOD1-G93A mice worsened motor decline and amplified neuroinflammatory gene signatures (10). Conversely, in a C9orf72-ALS model, reduction of the load of gut microbes (with antibiotics) even enhanced inflammation and survival (13), suggesting ALS genetic subtypes can differ in microbiota interactions (20). Such complexity supports that a balanced, rather than zero, microbiome appears protective at least in the SOD1 model (10) (**Figure 3**).

### 3.3 Neuroactive metabolites

Gut bacteria produce neuroactive metabolites. A. muciniphila is unique: it degrades mucin to maintain the mucus layer and synthesizes nicotinamide, a precursor to NAD+ (10). Gavage with A. muciniphila in ALS mice enhanced disease progression, an effect linked to elevated nicotinamide levels; ALS patients themselves have decreased systemic and CSF nicotinamide, implying significance (10). Butyrate-producing bacteria are responsible for keeping colonic health and regulatory immunity (18), notably Faecalibacterium prausnitzii, Eubacterium rectale/E. hallii, Roseburia spp., Butyrivibrio spp., and Anaerostipes spp. (8, 13), and butyrate itself can cross the gut wall and suppress HDACs in microglia, causing a neuroprotective phenotype (18). Decreased blood butyrate levels were detected in one patient cohort (13). Numerous microbes of the gut synthesize neurotransmitters or precursors: for example, Bifidobacterium and Lactobacillus species are known as producers of GABA (4), while tryptophan-metabolizing bacteria control the availability of serotonin (4). Loss of such producers would shift the glutamate-GABA balance toward excitotoxicity (12). In PD models, Lactobacilli supplementation increases host brain GABA and serotonin (4); by analogy, we would expect that in ALS their loss could remove a crucial brake on excitatory drive (15).

### 3.4 Microglia and immunity

ALS motor neuron degeneration is linked to widespread microglial activation (1). Gut microbiota significantly modulates microglial phenotypes even in conditions of steady state (18). A recent SOD1-G93A mice study found that gut bacteria removal shifted microglia to a neurodegenerative profile with higher inflammatory gene expression and lower homeostatic markers before symptom worsening (10). For clarity, microglial ‘homeostatic markers’ here denote the conserved surveillant signature—exemplified by P2RY12 and TMEM119 surface/signature genes, along with core transcripts such as CX3CR1, SALL1, and HEXB—that decline as microglia transition to activated or disease-associated states (16, 24). Thus, endogenous microbiota generally inhibit ALS-relevant microglial neurotoxicity (10). Microbes also establish peripheral immunity: dysbiosis can enhance pro-inflammatory T cell populations or reduce regulatory cells, potentially worsening neuroinflammation (20). For instance, in other contexts A. muciniphila has been shown to elevate IL-17-producing T cells of the gut that can migrate to the brain (20), although the net effect may be dependent on cytokine milieu (20). In general, gut flora dysregulation has the potential to modify central and systemic immune circuits of significance to ALS pathogenesis.

### 3.5 Evidence from allied disorders

Analogical support for our model comes from other neurodegenerative diseases. In Alzheimer’s disease (AD) mice, A. muciniphila administration improves gut barrier function, lowers circulating endotoxin, and attenuates cerebral amyloid pathology (27, 28). In PD models (29), administration of probiotic Bifidobacterium and Lactobacillus strains lowers *α*-synuclein-induced neurodegeneration by augmenting mucus secretion and lowering neuroinflammation. These findings suggest that modulation of the gut microbiota can affect CNS disease in a positive manner. While ALS is exceptional in its genetics and selective motor neuron involvement, it does share downstream pathways (neuroinflammation, oxidative stress, excitotoxicity) that are plausibly modulated by the microbiome. For example, PGC-1*α* (a mitochondrial regulator) and the serotonergic system have been implicated in neuroprotection in ALS as well as other CNS disorders, and gut microbes can influence these pathways.

## 4 Discussion

### 4.1 Central hypothesis

We propose that ALS-linked gut dysbiosis selectively depletes key symbiotic taxa—i.e., *Akkermansia muciniphila*, *Bifidobacterium longum*, and *Lactobacillus reuteri*. In a healthy gut, these microbes ensure intestinal barrier integrity and regulate systemic inflammation. For example, *A. muciniphila* has been shown to preserve mucosal integrity, inhibit metabolic inflammation, and enhance GLP-1 secretion through ICAM-2-mediated signaling. Probiotic *Lactobacillus* and *Bifidobacterium* strains maintain intestinal health through enhanced tight junction proteins such as occludin and claudins (30, 31), while modulating host cytokine responses and gut permeability (32). In inflammatory models, treatment with *Bifidobacterium* restores expression of tight-junction proteins and reduces TNF-*α* and IL-6 levels (31). Similarly, *L. reuteri* protects gut barrier integrity by lowering post-antibiotic dysbiosis and inflammation in intestinal models (33).

Key to their therapeutic actions, these microbes produce neuroprotective metabolites. *A. muciniphila* is a significant microbial source of nicotinamide (vitamin B3), an NAD+ and NADP+ precursor vital for mitochondrial energy metabolism and neuronal repair (10). Oral *A. muciniphila* supplementation in SOD1-G93A ALS mice raises CNS nicotinamide concentrations, improves motor function, and postpones neurodegeneration (10). ALS patients show reduced systemic and CSF nicotinamide levels, corroborating this pathway’s translational significance (10).

Concomitantly, *B. longum* and *L. reuteri* ferment dietary fibers into short-chain fatty acids (SCFAs) such as butyrate and produce *γ*-aminobutyric acid (GABA), a critical inhibitory neurotransmitter (19). In SOD1 mouse models, probiotic administration containing these species elevates serum SCFAs, normalizes microbial diversity, and prolongs survival (15). GABA-producing *Lactobacillus* strains have been shown to increase gut epithelial barrier integrity and inhibit inflammation (19).

According to our hypothesis, depletion of these taxa in ALS reduces systemic nicotinamide, SCFAs, and GABA, destabilizing neuroimmune homeostasis. SCFAs regulate microglial maturation and promote anti-inflammatory phenotypes; their deficiency can push microglia toward a neurotoxic phenotype (24). Reduced microbial GABA production is implicated in cortical disinhibition, an early and key ALS feature (34). Convergent neurophysiology shows primary motor cortex hyperexcitability in ALS, including reduced short-interval intracortical inhibition (SICI), shortened cortical silent period, altered motor thresholds, and enhanced intracortical facilitation, often preceding or tracking early disease (1, 34). These findings align with reduced GABAergic inhibitory tone in cortical and model systems, supporting excitotoxic stress as a mechanistic link (21). Longitudinal studies highlight SICI and related measures as sensitive progression biomarkers, reinforcing cortical disinhibition as a disease hallmark rather than a single-study artifact (1, 34). In this context, Kiernan et al. synthesize cortical hyperexcitability as a core ALS feature and its clinicophysiological relevance (1), Blacher et al. connect gut-derived metabolites (e.g., nicotinamide, SCFAs) to neuronal resilience and microglial states (10), and Mazzini et al. discuss translational avenues leveraging microbiota to modulate excitability and inflammation (3).

Gut barrier integrity loss allows microbial translocation (e.g., LPS) into the bloodstream, potentially preconditioning neuroinflammation via immune activation (18, 20). Broad-spectrum antibiotic-induced dysbiosis increases neurodegenerative microglial gene expression and decreases survival in SOD1 mice (10, 14).

The model proposes ALS-associated microbiome alterations initiate a gut-to-CNS immune cascade: loss of beneficial microbes disrupt mucosal homeostasis, reduce regulatory T cell induction, and skew immunity toward Th17/Th1 polarization (3). Microbial LPS and metabolites activate gut dendritic cells and macrophages, promoting pro-inflammatory cytokines (IL-6, IL-17, IL-1*β*), which traffic to the spinal cord, engage glial cells, and induce neurotoxic responses (22). IL-17A, elevated in ALS patients, directly impairs motor neuron survival *in vitro* (22). This creates an inflammatory loop culminating in microglial activation and motor neuron injury.

In summary, we hypothesize that loss of *Akkermansia muciniphila*, *Bifidobacterium longum*, and *Lactobacillus reuteri* in ALS impairs intestinal barrier integrity, reduces neuroactive metabolites, and sustains neuroinflammation, driving cortical hyperexcitability and degeneration. Targeted therapies replenishing these microbes or metabolites could stabilize cortical inhibition and slow disease progression.

### 4.2 Controversial findings and issues

Not all studies uniformly support a simple dysbiosis-ALS link. Conflicting human microbiome trends and genetic model differences (e.g., C9orf72’s response to antibiotics differs from SOD1’s) indicate complexity (11, 35). Whether microbiome changes are causes or consequences of disease, given factors like dysphagia affecting diet, remains uncertain. Even secondary dysbiosis might contribute to a self-sustaining loop where neuroinflammation impairs gut function, further altering microbiota (11). Stringent, longitudinal, and interventional studies remain critical (4).

### 4.3 Future directions and testable predictions

Microbiome interventions included targeted probiotics (*A. muciniphila*, *B. longum*, *L. reuteri*) and fecal microbiota transplantations should be tested in ALS models for efficacy in restoring metabolites, improving gut barrier function, and modulating immune responses (4, 15). Clinical trials may assess safety, microbiome composition changes, and neurophysiological markers (2, 12).

Biomarkers such as SCFAs, nicotinamide, and immune profiles can facilitate patient stratification and monitor treatment response (11). Dietary and genetic interaction studies in humanized animal models may reveal mechanistic insights (2).

## 5 Immune dysregulation and neuroinflammation

### 5.1 T cell polarization

Dysbiosis drives a shift in the systemic T cell pool. ALS patients exhibit a pronounced Th1/Th17 bias and reduced regulatory T cell (Treg) function. Th17-related cytokines (IL-17A, IL-23) are elevated in ALS blood and CSF, and IL-17A has been shown to directly decrease motor neuron viability. Conversely, Tregs normally suppress neuroinflammation, and reduced Treg activity in ALS is associated with faster progression. In fact, early-phase trials of exogenous Treg infusions report safety and potential slowing of disease (36), underscoring the importance of Treg-mediated tolerance in ALS. We posit that gut dysbiosis perturbs the balance of Th17/Treg generation (for instance, via altered short-chain fatty acid levels), favoring pro-inflammatory T cells that infiltrate or signal into the CNS.

### 5.2 Gut-brain immune trafficking

Breach of the epithelial barrier permits translocation of microbial components (e.g. LPS) and recruitment of innate immune cells. Elevated LPS is documented in sporadic ALS, and experimental LPS (at physiological concentrations) disrupts intestinal tight junctions via TLR4/FAK/MyD88 signaling. This fosters local intestinal inflammation and systemic endotoxemia. Activated macrophages and dendritic cells secrete IL-1*β*, TNF-*α*, IL-6 and other cytokines. Importantly, these cytokines can traverse the blood-brain/spinal cord barriers through diffusion or active transport mechanisms, reaching glial cells. Astrocytes and microglia express receptors for IL-1*β*, TNF-*α*, etc.; their engagement triggers a neuroinflammatory program. For example, systemic IL-1*β* and TNF-*α* are known to induce reactive astrocytosis and augment microglial neurotoxicity. Thus, gut-originating signals effectively “prime” CNS immunity: pro-inflammatory cytokines produced in the periphery penetrate the CNS and stimulate local innate cells.

### 5.3 Microglial activation

Gut-driven immune signals ultimately bias microglial phenotype. The microbiota is a major determinant of microglial maturation and activation state. In homeostasis, the gut fosters a balanced microglial repertoire: dysbiosis drives microglia toward a chronic M1-like state, (with loss of homeostatic markers such as P2RY12 and TMEM119) (16, 24). Recent evidence shows that absence of a normal microbiome alters microglial transcriptomes and increases pro-inflammatory microglial subpopulations. In ALS, microglia adopt a disease-associated phenotype with sustained release of ROS and cytokines (IL-1*β*, IL-6, TNF-*α*). These M1 microglia contribute to motor neuron injury. In contrast, M2-like microglia (induced by signals such as IL-4/IL-10) are neuroprotective. We propose that loss of microbiota-derived modulatory signals (e.g. butyrate, bile acids) diminishes M2-promoting cues, reinforcing microglial neurotoxicity. Indeed, one study found that genetic ALS models with high inflammation showed upregulation of innate immune receptors (like RAGE) and release of pro-inflammatory exosomes from microglia. Thus, dysbiotic gut signals (via LPS, TLR4 and peripheral cytokines) catalyze a vicious cycle: activated microglia sustain neuroinflammation and accelerate ALS progression.

### 5.4 Epithelial-immune barrier integrity

Homeostatic crosstalk at the mucosal interface is disrupted in ALS. Gut microbes normally educate intestinal dendritic cells and intraepithelial lymphocytes to maintain tolerance (37). Dysbiosis can weaken tight junctions and mucus layers, as seen in ALS models with increased gut permeability. A leaky gut leads to constant immune stimulation by luminal antigens. Chronic mucosal inflammation may skew local immune cells toward inflammatory phenotypes (e.g. Th17 induction by segmented filamentous bacteria). Additionally, microbiota-derived metabolites (such as altered tryptophan/kynurenine ratios or reduced butyrate) can influence Treg/Th17 balance and dendritic cell activity. In ALS patients, stool analyses often reveal mucosal immune activation (e.g. elevated fecal IgA) consistent with barrier compromise. We hypothesize that barrier failure is a key initiating event: increased exposure to microbial products perpetuates systemic inflammation and sensitizes CNS glia.

## 6 Conclusion

In summary, we propose that the gut microbiota is a modifiable risk factor in ALS pathogenesis. This hypothesis integrates disparate observations—dysbiosis, barrier dysfunction, metabolic deficiencies, and neuroinflammation—into a coherent picture. It also positions ALS not only as a cell-autonomous neuronopathy but as a systemic disease modulated by the overall environment of the body (3, 4, 17). Given the rapid progression and current therapeutic dead-end, research into the gut-brain axis offers a thrilling translational route (2). If realized, microbiota-directed interventions (probiotics, prebiotics, fecal transplants, or microbial metabolites) could form a novel class of ALS treatments (15). We challenge the ALS research community to test these ideas in rigorous trials, leveraging interdisciplinary expertise. This microbiota-ALS hypothesis might energize the broad medical forum, unifying neurology and microbiome science to create a desperately needed breakthrough (4, 11).

## Data Availability

The original contributions presented in the study are included in the article. Further inquiries can be directed to the corresponding author.
